# Molecular Regulation of Paused Pluripotency in Early Mammalian Embryos and Stem Cells

**DOI:** 10.3389/fcell.2021.708318

**Published:** 2021-07-27

**Authors:** Vera A. van der Weijden, Aydan Bulut-Karslioglu

**Affiliations:** Max Planck Institute for Molecular Genetics, Berlin, Germany

**Keywords:** embryonic diapause, pluripotency, dormancy, metabolism, transcription, miRNA, signaling pathways, stem cells

## Abstract

The energetically costly mammalian investment in gestation and lactation requires plentiful nutritional sources and thus links the environmental conditions to reproductive success. Flexibility in adjusting developmental timing enhances chances of survival in adverse conditions. Over 130 mammalian species can reversibly pause early embryonic development by switching to a near dormant state that can be sustained for months, a phenomenon called *embryonic diapause*. Lineage-specific cells are retained during diapause, and they proliferate and differentiate upon activation. Studying diapause thus reveals principles of pluripotency and dormancy and is not only relevant for development, but also for regeneration and cancer. In this review, we focus on the molecular regulation of diapause in early mammalian embryos and relate it to maintenance of potency in stem cells *in vitro*. Diapause is established and maintained by active rewiring of the embryonic metabolome, epigenome, and gene expression in communication with maternal tissues. Herein, we particularly discuss factors required at distinct stages of diapause to induce, maintain, and terminate dormancy.

## Introduction

Five momentous periods characterize the storyline of most animal life: fertilization, embryonic development, juvenility, sexual maturation, and reproduction. The reproductive drive motivates all of these steps, with the ultimate goal of contributing an individual’s genes to the next generations. Numerous reproductive tactics are employed to maximize the fitness of the young. For example, red-sided garter snakes store sperm for up to 1 year after mating to adjust the timing of fertilization, and to allow post-copulatory sexual selection. Wallabies mate within 1 h after giving birth and always rear an offspring, ensuring that any lost pouch young can be replaced as quickly as possible. In developing embryos of many species, the precursors of oocyte and sperm, primordial germ cells, are put aside even before the early embryo has established major tissues for itself, which may reduce the likelihood of *de novo* mutations in the germline (Milholland et al., 2017).

Most fish and amphibians produce numerous offspring, of which only a small percentage survives juvenility. In contrast, mammals produce 1–15 offspring at once and look after them a considerably long time thereby increasing the chances of juvenile survival. For this reason, the mammalian investment in each offspring during gestation, lactation and further nurturing of the young is energetically and temporally expensive, for which the necessary resources may not exist at all times. From a species’ survival point of view, flexible adjustment of reproductive timing in response to metabolic and environmental restrictions could make the difference between survival and extinction. In more moderate circumstances, such flexibility enhances reproductive efficiency.

Fertilization initiates the progressive development of the embryo. Within the first few days of development, most mammalian embryos activate their genome for transcription, go through a few cell divisions, and organize into the first embryonic structure called the blastocyst. Embryonic and extraembryonic cells are already allocated, yet undifferentiated, in the blastocyst. Although it takes place *in vivo*, this pre-implantation period of embryonic development is self-sufficient, as the fertilized oocyte can proceed through the same developmental steps in a minimally enriched culture medium *ex vivo*. Once at the blastocyst stage, and if the uterus is receptive, the embryo implants and proceeds with further developmental steps including gastrulation and organogenesis. *Ex vivo* cultured blastocysts cannot sustain pluripotency under standard culture conditions and collapse within 2–3 days (Bulut-Karslioglu et al., 2016). Thus, blastocysts can only be cultured transiently in regular embryo culture medium. Taken together, mammalian early development proceeds through sequential steps coordinated by maternal and embryonic programs, and is largely unyielding to temporal adjustments of individual steps. However, many mammalian species can *delay* embryonic development at the blastocyst stage to adjust the timing of birth such that the offspring and the mother will have a better chance of survival during the energetically demanding lactational period and beyond. Here we focus on delayed development by inhibition of embryo implantation, also called delayed implantation or *embryonic diapause*. A few other species, such as some bats, can delay development after implantation, which is beyond the scope of this review. Importantly, many other vertebrates and non-vertebrates such as several species of nematodes, crustaceans, fish, and birds can also suspend development in accordance with environmental conditions. Although also referred to as diapause, non-mammalian embryos are commonly *paused* at more complex embryonic stages (e.g., with fully specified tissues in the annual killifish) compared to the blastocyst, and are thus largely out of the focus of this review. However, where appropriate, we discuss potentially common regulatory mechanisms between mammalian and non-mammalian embryonic diapause.

### Mammalian Diapause Is a Feature of the Blastocyst

Although the embryos of some species such as cow begin gastrulation before implantation (van Leeuwen et al., 2015; Pfeffer et al., 2017), the blastocyst is usually the stage at which mammalian embryos implant. Therefore, it is also the stage that diapause occurs in the absence of implantation. The embryo is only conducive to diapause at the blastocyst stage, and not earlier, even when diapause is induced experimentally *in vivo* and *ex vivo* (Bulut-Karslioglu et al., 2016; Renfree and Fenelon, 2017). Many mammalian embryos fail to develop beyond the cleavage stages due to genetic aberrations or other causes (van der Weijden and Ulbrich, 2020). Pausing of embryos at the blastocyst stage, and therefore after cleavage stages, would readily integrate this initial quality control, such that only successfully developing embryos would be kept dormant for further development (van der Weijden and Ulbrich, 2020).

Most mammalian (eutherian) blastocysts contain three different cell types: cells on the outside of the embryo, the trophectoderm (TE), are the precursors of the placenta; and cells on the inside of the blastocyst, the inner cell mass, are a mix of precursors of embryonic tissues (the epiblast, Epi) and the yolk sac (the primitive endoderm, PrE). Stem cells representing the three early embryo cell types can be derived from the blastocyst and cultured *in vitro*. Trophectoderm stem (TS) cells represent the trophectoderm layer and can be differentiated into distinct trophoblast cells (Tanaka et al., 1998). Embryonic stem (ES) cells represent the epiblast and can generate all embryonic cell types across the three germ layers, as well as germ cells, therefore are pluripotent (Evans and Kaufman, 1981; Martin, 1981). Extraembryonic endoderm (XEN) stem cells represent the PrE and can be differentiated into more committed endodermal cells representing the yolk sac (Kunath et al., 2005). For clarity, we will refer to the early embryonic cells (Epi, PrE, TE) as cell types, and their *in vitro* counterparts as stem cells. The three early embryo stem cell types have distinct transcriptional and epigenetic profiles *in vitro* (ES, TS, and XEN) and *in vivo* (TE, Epi, and PrE) (Blakeley et al., 2015). Thus, common and distinct molecular regulators may be required to induce and maintain diapause in the different embryonic cell types. Furthermore, signaling and crosstalk between the different cell types likely instruct the paused stem cell states. Technical and material limitations usually prevent separate investigation of TE, Epi, and PrE cells *in vivo*. Stem cell models are thus valuable tools to dissect mechanisms regulating the three lineages. Although stem cell lines have so far been established in several mammalian species, most notably from rodents and humans, it remains a challenge to establish stem cell models of most wildlife species (Stanton et al., 2019). Except for mink, many diapause species lack stem cell models to date (Sukoyan et al., 1992; Menzorov et al., 2015), challenging identification and validation of molecular and genetic regulators of embryonic diapause. Derivation and induction of stem cells benefit from mapping and understanding of the transcriptional networks controlling embryonic cell types. Current low-input and single-cell transcriptomics and chromatin accessibility mapping technology allows blueprinting of gene networks in single embryos and cells (Blakeley et al., 2015; Wu et al., 2016; Guo et al., 2017). Application of these techniques to diapause embryos will expand the understanding of diapause pathways. It could further allow stem cell derivation from blastocysts or reprogramming of somatic cells to pluripotency via introduction of transcription factors governing each lineage.

### Triggers of Diapause and Reactivation

**FIGURE 1:**
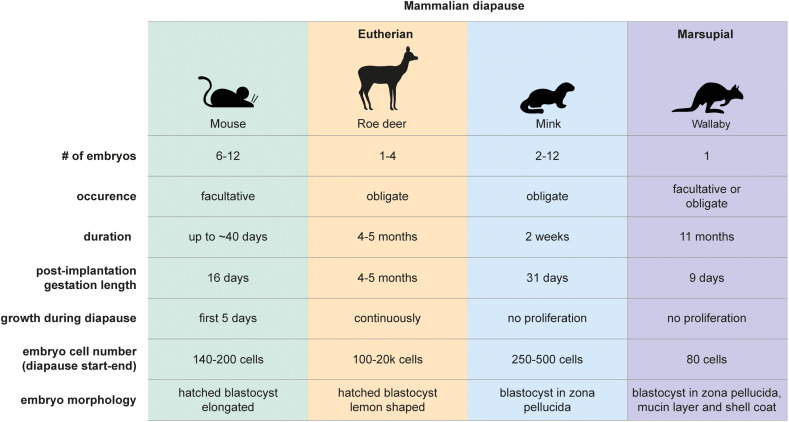
Mammalian diapause characteristics in the mouse, roe deer, mink, and wallaby. Embryo morphology, diapause duration, and embryo number differ between species. Diapause duration is independent of the length of post-implantation gestation. Diapause is triggered either seasonally (obligate) or due to lactational stress (facultative). Embryo reactivation is triggered by alterations in photoperiod (mink, wallaby, roe deer) or end of lactation (mouse, wallaby).

Diapause was first observed in the European roe deer (*Capreolus capreolus*) in 1854 (Bischoff, 1854). It is now known that over 130 mammalian species across eutherians and marsupials undergo diapause (Renfree and Fenelon, 2017; Figure 1). The duration of diapause is independent of gestational length and is rather a function of the prolongation required to optimally adjust the timing of birth (Renfree and Fenelon, 2017). Diapause is either obligate or facultative, the latter case being triggered by lactational stress. The American mink (*Neovison vison*) employs obligate diapause every mating season and adjusts the length of diapause according to the timing of mating (diapause length is 1–2 weeks) (Murphy, 2012). Exit from diapause is triggered by increasing daylight (photoperiod) following the March equinox in the northern hemisphere (Pilbeam et al., 1979; Murphy et al., 1981; Douglas et al., 1998). Blastocysts of the house mouse (*Mus musculus*) undergo diapause if lactation and pregnancy occur at the same time. Diapause regulation is most intensely studied in mice, due to the availability of *in vivo*, *ex vivo* and *in vitro* models and the feasibility of functional gene perturbations. Diapause can be experimentally induced in the mouse by surgical removal of ovaries (ovariectomy) at embryonic day E3.5 or by injection of estrogen antagonists (Weitlauf and Greenwald, 1968; Hunter and Evans, 1999), both of which counteract the estrogen surge prior to implantation. In addition to eliminating estrogen, progesterone supplementation is required to sustain the pregnancy. Mouse diapause can be sustained for up to 36 days and possibly longer *in vivo* (twice as long as gestation), however, embryo loss occurs over time (Arena et al., 2021). Exit from diapause is triggered by an increase in uterine receptivity that either occurs when lactation ends or can be experimentally induced by injection of estradiol. Mouse blastocysts can also be induced to enter a diapause-like paused state *ex vivo* via a few alternate methods for variable durations, including chemical inhibition of the cytoplasmic kinase mTOR (up to 30 days after blastocyst formation) (Bulut-Karslioglu et al., 2016), inhibition of the transcriptional regulator Myc (18 h) (Scognamiglio et al., 2016), and overexpression of the microRNA let-7 (up to 14 days) (Liu et al., 2020). These molecular regulators are further discussed below. Importantly, any *ex vivo* pausing method should be reversible and should not compromise the developmental potential of the blastocyst. Retransfer experiments in which paused, then released embryos are transferred to pseudopregnant surrogate female mice are necessary to test the developmental competence of experimentally paused embryos.

Marsupials also use diapause to adjust reproductive timing. Tammar wallabies (*Macropus eugenii*) generate one embryo per fertilization cycle, which undergoes both seasonal and lactational diapause for a remarkable 11 months (Renfree and Fenelon, 2017). After activation, marsupial embryos do not *per se* implant but loosely attach to the uterine wall, where they develop for about 26 days (Fenelon et al., 2017). The duration of diapause thus exceeds the duration of gestation by 10-fold in tammar wallabies. Wallaby diapause also differs from mouse and roe deer in that the blastocyst does not hatch out of its three embryonic coats (zona pellucida, mucin layer, and the shell coat) during diapause (Renfree and Fenelon, 2017). In contrast, mice, rats, and roe deer blastocysts have one embryonic coat (zona pellucida), out of which they hatch before diapause and eventual implantation. Additionally, marsupial blastocysts neither have an inner cell mass nor show differential staining of Epi, TE, or PrE markers in the blastocyst, suggesting that cell fate specification has not happened at this stage (Frankenberg et al., 2013). Exit from diapause in wallabies is triggered either by removal of the pouch young and its sucking stimulus, or by the onset of the summer solstice in the southern hemisphere (Renfree and Fenelon, 2017). Despite the occurrence of embryonic diapause in over 130 mammals, we do not have even a basic understanding of diapause duration and regulation in most species, including vulnerable species such as the panda bear.

### Are Embryos in Diapause Truly Dormant?

Dormancy represents a cellular state with reduced metabolic activity and little or no proliferation. For clarity, here we define dormancy as a complete loss of proliferation. We use the term “quiescence” only when evidence supports exit of cells from cell cycle. Embryonic diapause is characterized by a significant reduction in proliferation. But not all diapause embryos are truly dormant. About 10% of cells in the roe deer blastocyst proliferate throughout diapause, with the embryo expanding from 300 to 20,000 cells within 4 months (Rüegg et al., 2020). The inner cell mass proliferates less than the TE. The inner cell mass also undergoes morphological changes, from round to flattened to cyst to disc, indicating active restructuring and communication between the cells, even at the low rate of proliferation (Rüegg et al., 2020).

The mouse embryo cells reach near complete dormancy after 5 days in diapause. Kamemizu and Fujimori (2019) used the FUCCI model combined with staining of the proliferation marker Ki67 to investigate the cell cycle status of diapause mouse embryos over time and showed significant differences in the TE and ICM responses to diapause. While mural trophectoderm (opposite side to ICM) largely stops proliferating 1 day after induction of diapause, polar trophectoderm and the ICM gradually decrease proliferation over the next 4 days, reaching near-complete dormancy by diapause day 8.5 (D8.5, also called equivalent day of gestation 8.5, EDG8.5) (McLaren, 1968; Kamemizu and Fujimori, 2019). The sequence is reversed during exit from diapause, where the embryonic side (ICM and polar TE) activates before the mural TE. Prolonged diapause leads to a deeper dormant state which takes longer to activate. Upon activation and retransfer of EDG4.5 and EDG10.5 embryos, the authors found a 0.5–1 day delay in development of EDG10.5 embryos (Kamemizu and Fujimori, 2019).

The mouse embryo grows by about 140 cells and an estimated 4 times the volume during diapause compared to E4.0 blastocysts (Kamemizu and Fujimori, 2019). The epiblast and PrE do not grow, and, in contrast, can shrink by 40–50% (Batlle-Morera et al., 2008). As a result, the TE grows significantly during the course of diapause. As mural TE ceases proliferation by E4.5–E5.5, the polar TE should then be responsible for most TE proliferation. TE proliferation, together with potential stretching as evidenced by increased distance between TE nuclei, are likely responsible for the characteristic elongated shape of the mouse embryo in diapause. We note that there is great variability between mouse embryos of the same strain as well as between strains in terms of cell proliferation and epiblast size. This phenotypic variation may affect the consequent developmental competence of the embryos and needs to be taken into account when determining sample sizes in mouse diapause experiments to robustly identify novel diapause markers and regulators.

### Conservation of Diapause Potential in Non-diapausing Species

Whether diapause is conserved across mammals is an intriguing question. Eutherian blastocysts generally follow the same blueprint of pre-implantation development, although exact cell type-defining factors might vary between species (Blakeley et al., 2015; Nakamura et al., 2016; Petropoulos et al., 2016; Bernardo et al., 2018; Boroviak et al., 2018; Gerri et al., 2020). In this respect, more species might be capable of diapause than those actually employing it. To test whether non-diapausing species are responsive to dormancy triggers in the diapause uterus, interspecies transfer experiments have been performed. Embryos from two closely related species, the mink and the ferret (*Mustela putorius furo*) were transferred reciprocally (Chang, 1968). Ferret embryos showed delayed development and did not implant in the mink uterus under diapause, while normally diapausing mink embryos activated and implanted into the ferret uterus. An independent study showed sheep embryos undergoing diapause in the mouse uterus under diapause conditions, which upon activation and retransfer were able to give rise to live-born lambs (Ptak et al., 2012). These studies point to uterine control of diapause induction and maintenance and the conservation of diapause pathways, at least in mammals that have been studied. Although diapause is initially triggered by the non-receptivity of the uterus to an otherwise implantation-ready blastocyst, soluble uterine factors likely sustain the diapause state, as mouse and mink embryos in diapause do not remain dormant in basic culture medium lacking growth factors or relevant metabolites beyond a few days (Naeslund, 1979; Fenelon and Murphy, 2017). Therefore, the diapause uterine fluid is instructive in rewiring genetic pathways for maintenance of early embryonic dormancy.

### The Possibility of Diapause in Humans

A proven case of human diapause has never been documented. In naturally conceived pregnancies, precise determination of the exact time of fertilization or implantation is unlikely. Thus, if natural diapause exists in humans, it could only be detected by studying pregnancies following transfer of *in vitro* fertilized (IVF) embryos. Although sparse evidence shows that a large delay between transfer and implantation is possible (e.g., one case study shows a 5-week delay Grinsted and Avery, 1996), large-scale studies have not found such outliers. Naturally conceived human embryos implant 7–11 days after ovulation, although a range of 6–18 days was observed (Wilcox et al., 1999). Importantly, late implantation is associated with a higher rate of pregnancy loss. Late implantation, without a reduced cell proliferation of the embryo, may be caused by natural factors such as delayed uterine receptivity. However, xenobiotic factors such as those resulting from smoking also cause a higher rate of late implantation and pregnancy loss (Jukic et al., 2011). Taken together, there is currently no evidence that humans might use natural diapause as a reproductive strategy. It is important to note that, if diapause occurs in humans, it will most likely be triggered by lactational or nutritional stress. In this context, clinical studies present a challenge, since participants are very unlikely to experience such stresses. On occasions where mothers experience such stresses, e.g., during famines and droughts, reproduction timelines have not been analyzed. Moreover, the natural variation of gestation length in humans makes the detection of a potentially short diapause period challenging.

Similar to the ability of ferret and sheep embryos to undergo diapause as mentioned above, it is possible that human embryos might have retained or acquired a capacity for diapause. This possibility can only be tested *ex vivo* using surplus embryos donated by IVF patients. Many IVF surplus embryos develop suboptimally, there is genetic variation in the human population and there is naturally more variable developmental rates of human embryos compared to captive-bred species, making it challenging to achieve statistical power. Nonetheless, qualitative evidence can be obtained. Indeed, Liu et al. (2020) recently showed a slight delay in the development of human blastocysts upon treatment with extracellular vesicles carrying the microRNA let-7g (52% vs. 30% day 7 survival in treated vs. control embryos). Human blastocysts were also reported to undergo diapause when coated with a mucin-mimicking synthetic gel, however, these did not retain the characteristic blastocyst morphology (Canton et al., 2016). Taken together, this evidence suggests that human diapause, if it occurs, likely lasts just a few days.

### Why Is It Important to Understand the Regulation of Diapause?

In progressive embryonic development, pluripotency co-occurs with proliferation and proliferation regulates gene activity. Mouse ES cells clearly illustrate this relationship, where high proliferative capacity is linked to hyper-transcription and -translation, which in turn promote open chromatin through gene turnover of transcriptional and euchromatin modifiers (Bulut-Karslioglu et al., 2018). In contrast, maintenance of pluripotency in diapause does not depend on proliferation. The diapause epiblast maintains naïve pluripotency networks and at the same time presents a distinct and largely suppressed transcriptional profile in response to altered cell proliferation (Boroviak et al., 2015). As such, diapause offers a unique model to dissect pluripotency and proliferation networks.

In addition to preserving the first three cell types in the embryo over longer periods, diapause might also enhance pluripotency. Indeed, the first ES cells (mouse) were derived from diapause embryos (Evans and Kaufman, 1981) and comparative analyses has shown that diapause embryos more efficiently gave rise to ES cells for the initial strain employed (129) as well as hitherto refractory strains (Brook and Gardner, 1997). The increased efficiency cannot be explained by increased embryo size, since epiblasts were extracted for ES derivation and later studies showed that the epiblast cell number does not increase, and contrarily may decrease during diapause (Batlle-Morera et al., 2008). Thus, embryonic dormancy likely enhances the ability to give rise to ES cells. Whether this effect is due to rewiring of transcriptional and epigenetic landscapes or by other means is unclear to date. An intriguing possibility is that potentially enhanced DNA repair during diapause might in several species enable the emergence of a healthier embryo upon diapause exit. Indeed, DNA repair proteins are expressed at higher levels during killifish diapause (Wagner and Podrabsky, 2015; Hu et al., 2020), but whether repair activity is enhanced in killifish and mammals during diapause needs to be further investigated. Enhanced autophagy and lower oxidative damage may be two alternative mechanisms that increase the fitness of the diapause embryo (see below). The above outlined potential benefits might result in a higher developmental potential of individual diapause epiblasts, however, further studies directly addressing its developmental capacity e.g., via chimera formation are required.

Prolonged diapause, on the other hand, may compromise the fitness of the embryo. In mice, fewer diapause embryos (Arena et al., 2021) and resulting fetuses (Weitlauf and Greenwald, 1968) are recovered with longer duration of diapause. Also, *ex vivo* survival of embryos in a diapause-like state diminishes over time (Bulut-Karslioglu et al., 2016; Liu et al., 2020). Metabolic restrictions, maternal or embryonic pathways may underlie diminishing embryo survival. Understanding metabolic and genetic regulation of diapause is thus critical to overcome the embryo lethality or compromised developmental competence. Under culture conditions tailored to species-specific requirements, diapause could be induced and maintained for longer periods *ex vivo*. Artificial reproductive technology would greatly benefit from such progress, especially in wildlife and captive-bred species for which cryopreservation is either not adaptable or compromises embryo fitness (Wauters et al., 2020). Prolonged blastocyst maintenance would also extent the time window for genetic selection or manipulation of embryos.

Embryo studies allow identification of transcriptional networks critical for survival of each cell type. The required transcription factors (TF) or cytokines can then be engineered and utilized to derive ES or extraembryonic stem cells or to reprogram somatic cells (Williams et al., 1988; Takahashi and Yamanaka, 2006). Some key factors enabling generation of pluripotent stem cells are only required in diapause and not in proliferative blastocysts, suggesting that pluripotency maintenance and establishment may be regulated by non-overlapping mechanisms. For example, leukemia inhibitory factor (LIF), a cytokine that allows maintenance of ES cell pluripotency (Smith et al., 1988; Williams et al., 1988), is dispensable in proliferative blastocysts (Stewart et al., 1992). Although the LIF pathway is not strictly necessary for ES derivation or maintenance *in vitro* (Ying et al., 2008), knockout of the LIF receptor component gp130 results in loss of the pluripotent epiblast during prolonged diapause (Nichols et al., 2001), providing a clear example of an *in vitro* pluripotency maintenance factor with physiological roots in diapause. Unraveling diapause networks may thus enable generation of ES cells from species with no established ES cell models.

### Other Phenomena With a Regulatory Basis Potentially Similar to Diapause

Dormancy-activation cycles underlie stem cell function in many somatic tissues, such as in the hematopoietic and mesenchymal systems (Marescal and Cheeseman, 2020). Although tissue-specific stem cells are not pluripotent and have transcriptional networks tailored to the tissue type and activation cues, dormancy and its reversibility in embryonic and adult tissues may share a regulatory basis. Furthermore, dormant cancer cells, which usually arise as a result of therapy resistance, also show similarities to diapause embryo cells (Dhimolea et al., 2021; Duy et al., 2021; Rehman et al., 2021). For example, metabolic profiles of dormant stem cells are remarkably similar across tissues and species, with globally decreased oxidative phosphorylation and an increased dependency on lipolysis (Kinder et al., 2010; Singh et al., 2016; Marescal and Cheeseman, 2020). Similarly, autophagy is enhanced in diapause cells, as well as in tissue stem cells and in dormant cancer cells (Lee et al., 2011; Bulut-Karslioglu et al., 2016; Hen and Barkan, 2019). The cytoplasmic kinase, mTOR is a major growth regulator that promotes proliferation in virtually every tissue (Laplante and Sabatini, 2012). Inhibition of its activity induces diapause in mouse embryos (Bulut-Karslioglu et al., 2016) and is necessary for tissue stem cell dormancy, as evidenced by loss of tissue stem cell pools in hyperactive mTOR mutants (Kharas et al., 2010; Zhang et al., 2015; Hu et al., 2017). Diapause studies may thus not only unravel developmental pathways, but also increase our understanding of tissue stem cell biology and cancer dormancy. The ability of stem cells to tolerate and adjust to various stressors in diapause could signify their capacity to resist to other stressors such as bacterial infections in *C. elegans* that the embryo might encounter during development (Ren and Ambros, 2015).

## Molecular Regulation of Diapause

### Uterine Receptivity and Hormonal Regulation of Diapause

Seasonal and lactational diapause are hormonally regulated by a number of hormones including prolactin, estrogen, and progesterone, acting in different manners in different taxa (Renfree and Fenelon, 2017). Here we summarize the main hormonal changes leading to diapause and reactivation of the early mammalian embryo.

**FIGURE 2:**
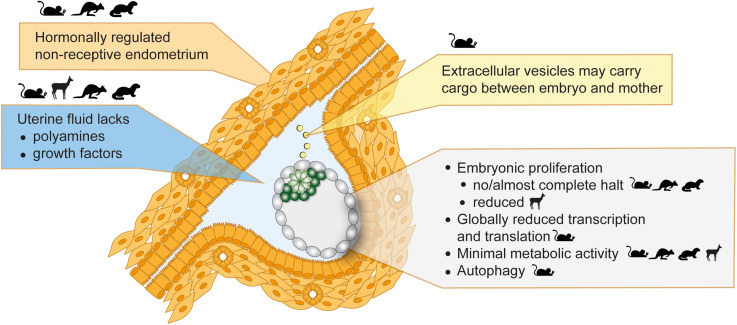
Inducers and features of embryonic diapause. Seasonal variations in photoperiod or lactational stress lead to hormonal alterations. The uterus then fails to establish a receptive state, which blocks implantations and induces diapause. Uterine fluid and extracellular vesicle composition is altered in diapause. The embryo in diapause shows minimal metabolic activity, globally reduced transcription and translation, and diminished or reduced proliferation. Depicted in the figure is a mouse embryo. Parent species where evidence supportive of each statement was derived are shown as icons.

Once at the blastocyst stage, the mammalian embryo is ready to implant into the uterine wall. Normally, a glycoprotein layer called mucins covers the uterine surface and acts as a barrier to implantation due to its anti-adhesive property (Carson et al., 1998). In mice, the estrogen surge on day 4 and, more importantly, the continuously high progesterone levels lead to temporary stripping of the mucin layer and provides a window of implantation (Surveyor, 1995). Lactation-induced decrease of gonadotrophin release by the pituitary gland and consequently lower estrogen levels cause a delay in embryo implantation (Whitten, 1955). Experimentally, surgical removal of ovaries or injection of estrogen antagonists reduce estrogen levels and induce diapause in mice (McCormack and Greenwald, 1974; Hunter and Evans, 1999). High progesterone levels are required throughout diapause to sustain the pregnancy. The embryo remains in close proximity to the uterine wall throughout diapause, in fact it localizes to pockets of uterine tissue called crypts in the implantation position (Figure 2), with the mural TE more proximally located to the uterus in the mouse (in contrast, the human embryo implants from the polar side) (Kamemizu and Fujimori, 2019). This positioning may enable close communication between the maternal tissue and the embryo via diffusible factors in the uterine fluid or via extracellular vesicles. Diapause is terminated *in vivo* upon decrease of lactation-induced prolactin followed by an increase in estrogen. Experimentally, injection of estradiol terminates diapause. The diapause mouse embryo activates within 12 h of the estrogen surge, although precise time of activation may vary depending on the length of diapause (Kamemizu and Fujimori, 2019).

In tammar wallaby, diapause is induced and maintained by inhibition of the corpus luteum-secreted progesterone via high plasma prolactin levels (Hinds, 1989; Hinds and Tyndale-Biscoe, 2012). Suckling of the pouch young or photoperiod-induced melatonin alterations control plasma prolactin levels (Shaw and Renfree, 1984). Between 48 and 72 h post decrease of prolactin levels either after the summer solstice or upon loss of the pouch young, the corpus luteum increases the secretion of progesterone, which leads to embryo reactivation and eventual implantation on day 17. The delayed reactivation of the embryo might be due to slow diffusion of activating factors through the embryonic shell coat.

The mink differs from mouse and wallaby in that neither prolactin nor progesterone is highly secreted during diapause (Møller, 1973; Douglas et al., 1994). Mink diapause is largely controlled by photoperiod-mediated changes in nocturnal melatonin levels. Following the March equinox, decreased melatonin levels lead to an increase in prolactin, progesterone secretion from the corpus luteum, endometrium receptivity, and embryo reactivation 3 days later (Pilbeam et al., 1979; Murphy et al., 1981; Stoufflet et al., 1989).

The failure of the uterus to establish a receptive state under the influence of upstream hormonal regulation is likely the principal cause for the initiation of diapause (Figure 2). This notion is further supported by genetic studies. LIF is secreted by the uterine glandular epithelium cells in response to estradiol and is necessary for implantation (Stewart et al., 1992). Maternal LIF knock-out (KO) results in non-implanted blastocysts with morphological features of diapause (Stewart et al., 1992). LIF expression correlates with the implantation window also in mink, Western spotted skunk, and humans (Song et al., 1998; Hirzel et al., 1999; Aghajanova, 2004). Although low LIF levels correlate with failed implantations in humans, a requirement for LIF in human implantation has not been proven (Aghajanova, 2004). Other important regulators of implantation and diapause are the muscle segment homeobox genes *Msx1* and *Msx2* (Daikoku et al., 2011). Msx genes are repressed directly by LIF, thus are highly expressed in the uterine luminal and glandular epithelial cells during diapause (mouse, mink, wallaby) and downregulated for implantation (Cha et al., 2013). Mouse diapause can be induced but not maintained in Msx KO mothers (Cha et al., 2013). However, whether Msx upregulation is sufficient to induce diapause is unclear and inducible overexpression in the uterus is necessary to prove causality. Taken together, uterus non-receptivity suffices to trigger diapause, however, additional factors are likely necessary to maintain prolonged pausing.

### The Uterine Microenvironment

The uterine fluid comprises of growth factors, soluble metabolites and extracellular vesicles, and serves as a communication medium between the embryo and the mother. Uterine fluid composition, i.e., free amino acids, glucose, lactate, and pyruvate concentrations, is tailored to the developing embryo (Harris et al., 2005), indicating stage-specific metabolic requirements across species, e.g., cattle (Forde et al., 2014), sheep (Gao et al., 2009), mice (Kelleher et al., 2016), and pig (Kim et al., 2013). Metabolite uptake is also regulated in a stage-specific manner by altered expression of metabolite transporters (Winkle, 2001). During diapause, the embryo stays in close proximity to the uterus and responds to diffusible factors such as LIF (Nichols et al., 2001). The uterine tissue, uterine fluid, and the embryo have been separately studied to identify molecular regulators of diapause. Reactivation from diapause is clearly associated with increased soluble growth factors in the uterine fluid, e.g., epidermal growth factor (EGF) and heparin-binding EGF (HBEGF) in the tammar wallaby (Fenelon et al., 2017), together with increased expression of growth factor receptors in the blastocyst, e.g., EGFR increase in mouse 12–24 h after estradiol injection (Hamatani et al., 2004). The absence of growth factors does not induce diapause in *ex vivo* cultured embryos, indicating that growth factor pathways are more relevant for reactivation.

Absence of certain metabolites do induce or maintain diapause, as shown by delayed activation of diapause mouse blastocysts *ex vivo* in the absence of glucose, and by delayed implantation of mink and mouse blastocysts *in vivo* in the absence of polyamines (Naeslund, 1979; Fenelon and Murphy, 2017). Polyamines are synthesized from ornithine, arginine, proline, and methionine by ODC1 downstream of mTOR in response to prolactin and play important roles in reproductive physiology by regulating endothelial cell growth and proliferation, cell migration, and via antioxidant functions (Lenis et al., 2017). Chemical inhibition of uterine ODC1 induces diapause in both mink and mouse blastocysts and uterine ODC1 is upregulated upon reactivation (Lefèvre et al., 2011b; Fenelon and Murphy, 2017). Likewise, polyamine abundance, i.e., less spermine, more spermidine and putrescine, likely increase upon reactivation in the uterine fluid of roe deer (van der Weijden et al., 2019). Other diapause-associated microenvironmental changes include adhesion- and cell cycle-related proteins, further corroborating maternal control over embryo attachment and proliferation (Lefèvre et al., 2011a; Martin et al., 2016; van der Weijden et al., 2019).

### Metabolic Profile of the Diapause Embryo

Similar to other dormant cells, the diapause embryo presents a significantly lowered metabolic rate with altered usage of metabolic pathways (Naeslund et al., 1980; Lee et al., 2011; Hussein et al., 2020; Sousa et al., 2020). The diapause mouse ICM is metabolically more quiescent compared to TE, suggesting cell type-specific regulation of metabolic activity (Houghton, 2006). In general, reduced oxidative phosphorylation, structurally altered mitochondria and increased autophagy characterize the diapause metabolism (Naeslund et al., 1980; Lee et al., 2011; Hussein et al., 2020; Sousa et al., 2020). Reactivation from diapause results in activation of mitochondria in the mural TE, as evidenced by increased mitochondrial membrane potential (Fu et al., 2014). However, the role of glycolysis is less clear, with studies reporting either increased or decreased glycolysis downstream of mTOR inhibition in ES cells and in diapause embryos (Fu et al., 2014; Boroviak et al., 2015; Hussein et al., 2020; Sousa et al., 2020). Reactivated mouse embryos display a rapid increase in expression of glycolytic pathway genes (Fu et al., 2014) as well as pyruvate and glucose uptake (Spindler et al., 1996). Reactivation is delayed in the absence of glucose (Naeslund, 1979) pointing to the critical role of glycolysis in exit from diapause (Spindler et al., 1996). The glutamine transporter Slc38a1 is also upregulated in the ICM during diapause and inhibition of it is detrimental to the embryos capacity to pause (Hussein et al., 2020).

Although RNA, protein, and metabolome profiling provide insights into metabolic pathway usage, real-time measurement of metabolic activity via colorimetric assays or the Seahorse system has the potential to provide more direct evidence of metabolic activity. However, these assays often require a large number of cells and therefore direct metabolic characterization of embryos remains challenging.

Dormant stem cells across tissues and species utilize lipid reserves for maintenance (Kinder et al., 2010; Singh et al., 2016). Recently, lipids have been shown to be a major energy source during diapause as well. Free fatty acids and phospholipid phosphatidylcholine are in greater abundance in diapause blastocysts compared to proliferating embryos, suggesting active lipolysis (Hussein et al., 2020). The lipid content of the mouse oocyte is relatively low compared to other species (4 ng compared to 161 ng in the pig, 63 in cows, and 89 in sheep) (Loewenstein and Cohen, 1964; McEvoy et al., 2000; Sturmey and Leese, 2003). Nonetheless, removal of lipid droplets from mouse zygotes impairs the survival of the diapause blastocysts, indicating active utilization during diapause (Arena et al., 2021). This functional evidence is supported by molecular analysis of different lipid species during diapause, which found depletion of neutral lipids usually enriched in lipid droplets (e.g., triacylglycerol) and increase in processed intermediates such as phosphatidylcholines and fatty acids (Hussein et al., 2020). Intriguingly, and contrary to this finding, fatty acid oxidation was shown to counteract a naturally arising quiescent subpopulation in mouse ES cells, with inhibition of it increasing the G_0_ population from 4 to ∼17% (Khoa et al., 2020).

The metabolic profile of a cell not only determines energy availability and levels of cellular building blocks, it also affects the epigenome, genome integrity, and cellular fitness. Preimplantation development takes place in a hypoxic environment, with intrauterine oxygen levels ranging from 1.5% in the rhesus monkey to 5.3% in rabbits and hamsters (Fischer and Bavister, 1993). The low-capacity oxidative phosphorylation mandated by hypoxia is further lowered during diapause, which may result in less oxidative DNA damage in the diapause embryo (Houghton, 2021). In addition, existing DNA damage may be more efficiently repaired during diapause. Cells turn to autophagy when other energy sources do not suffice to sustain the energy needs or reduced uptake or metabolism do not allow the use of existing resources. In addition to providing energy, autophagy also clears out defective organelles, mostly mitochondria, which is more prone to oxidative DNA damage compared to genomic DNA. Increased autophagy is a common feature of diapause (Lee et al., 2011; Hu et al., 2020) and dormancy and may in this way enhance cellular fitness. Fu et al. (2014), however, showed that mitochondria numbers remain constant during diapause. Thus, how autophagy contributes to overall diapause metabolism remains unclear. In contrast to its short-term benefits, ongoing autophagy could, in the long-term, compromise embryo health if organelles are depleted below a threshold required to sustain the embryo. Indeed, mouse embryos that have been diapause longer than 10 days resulted in fewer fetuses, potentially due to poorer development of fetal placenta vessels (Weitlauf and Greenwald, 1968). In this sense autophagy may not be an important factor in cell nutrition in species with long periods of diapause such as the tammar wallaby (Renfree and Fenelon, 2017).

### Cell Cycle

Dormancy mandates alterations in cell cycle progression and, indeed, cell cycle related genes are among the most altered between dormant and activated blastocysts. Evidence supports p21-mediated cell cycle control at the G1/S checkpoint, thereby retaining cells in the G0/G1 phase and reducing DNA replication (Hamatani et al., 2004; Kamemizu and Fujimori, 2019). Diapausing killifish cells similarly arrest at G0/G1, and upon activation immediately enter the S phase (Dolfi et al., 2019). In mice, it has been suggested that cell cycle arrest is estrogen-mediated and thus controlled maternally via downregulation of the tumor repressor Brca1 and upregulation of the anti-proliferation gene Btg1 (Hamatani et al., 2004).

In mouse ES cells, induction of a paused-like state by inhibition of mTOR reduces the proliferation rate while distribution of cells among the stages of the cell cycle is only modestly altered, suggesting that paused-like cells proceed through the cell cycle, albeit at a very slow pace (Bulut-Karslioglu et al., 2016). In contrast, Myc inhibition, which also leads to a diapause-like state, enriches G_0_/G_1_ cells and depletes the S phase (Scognamiglio et al., 2016). Although the latter cell cycle profile is closer to true dormancy, the Myc-inhibited cells cannot be sustained longer than a day. These findings suggest that *in vivo* and *in vitro* regulation of diapause might be distinct and warrants further studies on cell cycle control in paused pluripotency or embryonic stem cell dormancy.

## Genomic Regulation

Cell cycle and metabolism rewiring appear to be hallmarks of diapause, as expected from a largely dormant state. Yet, distinguishing causal and consequential alterations in diapause remains a challenge. Several RNA and protein profiling studies have mapped gene expression changes during diapause to identify altered pathways and to isolate inducers of diapause and reactivation. Here we outline in detail the recent advances in genomic regulation of diapause. We note that functional perturbations are necessary to show causality. In addition, we caution the reader to take the following critical confounding factors into account when applicable: (1) significant inter-embryo heterogeneity in diapause response, as evidenced by variable durations of pausing *in vivo* and *ex vivo*, may conceal changes if pooled embryo profiling is performed, (2) whole-blastocyst analysis may prevent identification of Epi-, TE-, and PrE-specific regulators, (3) static analysis (i.e., comparing a single time point vs. control) likely misses dynamically altered pathways, and (4) gene expression levels cannot be precisely mapped, due to global reductions in RNA and protein levels, unless exogenous spike-in controls are used. Despite these complications, exciting new insights into genomic regulation have illuminated diapause biology in the recent years.

### Chromatin Rewiring in Diapause: Cause or Consequence?

Epigenetic marks, such as post-translational modifications of histones, DNA and RNA, together with TFs and chromatin remodelers, regulate gene activity. Epigenetic modifications either control or result from transcriptional activity. Irrespective of causality, highly transcribed or repressed genes show characteristic histone modifications, e.g., histone acetylation is associated with high transcriptional output (Bannister and Kouzarides, 2011). Additionally, the chromatin state is linked to metabolism, as for example the citric acid intermediate α-ketoglutarate affects DNA and histone methylation levels (Donohoe and Bultman, 2012). As such, chromatin rewiring is an expected outcome of diapause, although a detailed characterization is missing. Chromatin of ICM cells is particularly sensitive to diapause and shows depletion of histone acetylation such as H4K16ac (Bulut-Karslioglu et al., 2016). Interestingly, knock-out of MOF, the enzyme that acetylates H4K16, increases the naturally-occurring dormant subpopulation of ES cells observed in the study, suggesting that depletion of histone acetylation could induce pausing by reducing global transcriptional output (Khoa et al., 2020). On the contrary, inhibition of histone acetyltransferase activity via chemical inhibitors does not suffice to pause mouse blastocysts *ex vivo* (Bulut-Karslioglu et al., 2016).

Transcriptional activity reduces the abundance of heterochromatin (Ahmad and Henikoff, 2001), therefore global reductions in transcription and associated histone acetylation in diapause may result in abundant heterochromatin. In agreement, electron microscopy analysis of diapause vs. active blastocysts revealed more condensed nucleoli and abundant heterochromatin in Epi and TE cells, which decondensed within 12 h of reactivation (Fu et al., 2014). Polycomb-mediated H3K27me3, which represses transcription, represses key metabolic and developmental genes during killifish diapause (Hu et al., 2020) and may also play a role in maintaining diapause in mammals. Although most of the genome is silent and potentially heterochromatinized during diapause, the diapause epiblast retains naïve pluripotency features (Boroviak et al., 2015). Additionally, a subset of genes is upregulated and possibly induces or maintains dormancy (Hamatani et al., 2004; Bulut-Karslioglu et al., 2016). The regulatory pathways by which such genes escape silencing is a question that invites attention in the coming years.

### Transcription and Translation Are Globally Reduced During Diapause

Inhibition of cellular growth and proliferation during diapause leads to global reductions in transcription and translation, resulting in smaller cell size. Paused-like ES cells have 2–4-fold less RNA per cell and are smaller than proliferative ES cells (Bulut-Karslioglu et al., 2016). Dormant cells across different systems, e.g., tissue stem cells, are smaller than their activated counterparts and have reduced transcriptional and translational output (Rodgers et al., 2014). The impact of reduced transcriptional and translational capacity is particularly relevant for the imminent expansion of the epiblast after implantation. Pluripotent cells of the late epiblast are among the most rapidly proliferating cells, with one cell division taking ∼5–6 h (Snow, 1977). This rapid proliferative rate mandates prolific transcription and translation. In proliferative ES cells, levels of transcription and associated chromatin modifications such as histone acetylation are acutely responsive to translational output, which is in turn correlated with transcriptional output (Schwanhäusser et al., 2011; Percharde et al., 2017; Bulut-Karslioglu et al., 2018). Reactivation of the diapause embryo, combined with the fast proliferative rate of the early post-implantation embryo, requires a major ramp-up in transcriptional and translational output. How the transcription-translation cycle builds back to prior levels and the associated chromatin regulation is an area of interest for future studies. In other situations requiring an accelerated transcriptional response, e.g., signal-induced transcription at heat shock or hormone-responsive genes (Sawarkar et al., 2012), pre-loaded RNA polymerase and chromatin modifiers enable rapid activation. Similar regulation may be utilized in diapause, as mouse embryos reactivate within 12 h after release from diapause (Kamemizu and Fujimori, 2019). In addition, post-transcriptional and post-translational mechanisms such as miRNA activity complement nascent regulation to achieve repression and on-demand activation of proliferative pathways.

### Transcriptional Networks and Cellular Signaling in Diapause

**FIGURE 3:**
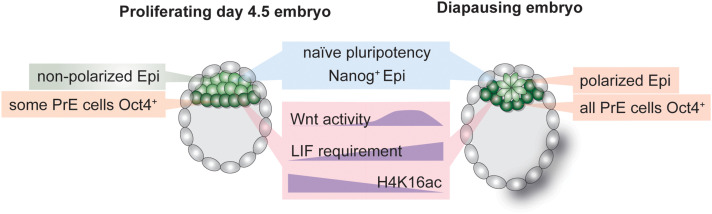
Hallmarks of the inner cell mass (ICM) of normal and diapause blastocysts. Establishment and maintenance of diapause entails alterations in cellular signaling pathways, transcriptional networks and the chromatin state. The epiblast retains naïve pluripotency during diapause but is polarized due to transient Wnt pathway activity. LIF is required to sustain the epiblast throughout diapause. The transcription-associated histone H4K16 acetylation is depleted in diapause ICM.

Stem cell identity is governed by transcriptional networks downstream of cellular signaling pathways (Young, 2011). Altered signaling pathway activity defines transcriptional outputs and can result in cell state or fate switches. In the mouse, pluripotency is maintained through combinatorial activity of master TFs such as Oct4, Klf4, Esrrb, Sox2, and Nanog under the control of signaling pathways such as Fgf/Mek/Erk, Wnt, or LIF/Jak-Stat. Long-term pluripotency maintenance during diapause entails distinct signaling pathway activity as compared to only transient regulation in proliferative blastocysts. For instance, although the cytokine leukemia inhibitory factor (LIF) is expressed in the blastocyst (Nichols et al., 1996), it is dispensable for early embryo development (Stewart et al., 1992). It is, however, required to maintain pluripotency during diapause, as gp130 (LIF receptor component) knock-out embryos lose the epiblast during diapause (Nichols et al., 2001). ES cell pluripotency is alternatively maintained through inhibition of the differentiation-promoting Mek/Erk pathway and enhancement of Wnt pathway activity (Ying et al., 2008). Wnt activity is minimal in the early embryo but is increased during diapause (Boroviak et al., 2015), illustrating another case where prolonged pluripotency maintenance in ES cultures has physiological roots in diapause. In general, naïve pluripotency networks are intact in the diapause epiblast, indicating active maintenance of pluripotency despite global transcriptional silencing (Boroviak et al., 2015). However, the effect of diapause on PrE- and TE-specific transcriptional networks is unclear. Technical challenges in dissecting Epi, PrE, and TE cells in the blastocyst so far prevented comparative studies of stem cell networks and crosstalk between the three cell types in the diapause blastocyst. Of note, although PrE is clearly defined in the diapause blastocyst, observations suggest altered transcriptional networks, e.g., all Gata4^+^ PrE cells were shown to retain Oct4 expression, in contrast to only a subset of Gata4^+^/Oct4^+^ cells in normal mouse blastocysts (Batlle-Morera et al., 2008; Figure 3). ICM cell numbers decrease by half (Epi) and 40% (PrE) during diapause (Batlle-Morera et al., 2008). Thus, the remaining ICM cells during diapause may be selectively retained based on their transcriptional state, allowing them to express both Gata4 and Oct4, or transcriptional rewiring may override previous separation of Gata4^+^ and Oct4^+^ in a subset of cells. Mouse trophectoderm shows signs of apoptosis during diapause, and its deterioration may underlie embryo loss in prolonged diapause (Bulut-Karslioglu et al., 2016). Thus, whether TE identity and transcriptional networks remain uncompromised is unclear. Species in which a single embryo experiences an extremely long diapause, such as the tammar wallaby, likely safeguard cellular potency via multiple redundant mechanisms, as the loss of the single embryo would have significant consequences on the species’ survival. Interestingly, wallaby blastocysts are composed of a monolayer of cells without an ICM, which may underlie their increased resistance to prolonged pausing.

A prominent pathway in controlling embryonic diapause in mice is the PI3K/mTOR pathway. mTOR promotes cell growth, and thus proliferation, via direct control over translation, metabolism, and transcription (Laplante and Sabatini, 2012). It acts as a cellular rheostat to adjust cellular growth to the availability of nutrients and energy. Modulation of mTOR activity is necessary for dormancy-activation cycles in adult stem cells, as hyperactive mTOR results in loss of stem cell pools (Kharas et al., 2010; Zhang et al., 2015; Hu et al., 2017). Similar to dormant tissue stem cells, the mTOR pathway is downregulated in the diapause epiblast, suggesting that the PI3K/insulin/mTOR axis governs dormancy decisions in mammals (Boroviak et al., 2015). Importantly, inhibition of mTOR activity induces diapause in mouse blastocysts *ex vivo* for up to 30 days (Bulut-Karslioglu et al., 2016). This diapause-like state can also be applied to ES cells in culture via treatment with catalytic mTOR inhibitors. The paused-like ES cells show characteristics of diapause such as a reduced metabolic rate, globally downregulated transcription and translation, and a transcriptional signature reminiscent of the diapause epiblast. Establishment of such *in vitro* diapause-mimicking stem cell culture conditions is critical to overcome technical and material limitations in embryo studies and for detailed mapping and functional investigation of regulatory networks.

Although translational inhibition is one of the most prominent consequences of mTOR inhibition, inhibition of translation does not suffice to induce diapause *ex vivo* (Bulut-Karslioglu et al., 2016), suggesting that combinatorial control downstream of mTOR inhibition drives cellular adaptation to diapause. A major regulator that controls global transcription as well as specific gene networks is TF Myc (Kress et al., 2015). Myc is a component of the pluripotency network and is also functional across somatic tissues (Kress et al., 2015). Myc activity is reduced in diapause (Boroviak et al., 2015) and its inhibition can induce a diapause-like state for short periods (18–24 h in mouse blastocysts) (Scognamiglio et al., 2016).

### Wnt Pathway Activity and the Interplay With Cellular Polarization

In contrast to reduced mTOR and Myc signaling, Wnt activity is transiently increased during diapause in the epiblast (Fan et al., 2020). The Wnt pathway is stimulated via multiple ligands (Wnt1-16 in mice and humans with variants, in total 19 genes) and can assume conflicting roles in promoting pluripotency or differentiation in a cell type specific and context-dependent manner (ten Berge et al., 2008, 2011). Wnt is also an upstream regulator of cellular polarization, thus spearheading epithelial-mesenchymal transition in gastrulation and body patterning. The ICM of the pre-implantation embryo comprises of unpolarized cells (Rivera-Pérez and Hadjantonakis, 2014). Immediately after implantation the ICM polarizes, assumes a rosette-shaped pattern, creates a lumen and undergoes morphological transformation to generate the classical “egg cylinder” of the mouse post-implantation epiblast (the post-implantation morphology is species-dependent, e.g., in human the post-implantation epiblast forms a disc shape) (Rivera-Pérez and Hadjantonakis, 2014). Morphological changes in the epiblast correspond to progression of the embryo along the pluripotency spectrum, i.e., from rosette (Neagu et al., 2020) to formative (Kinoshita et al., 2021) to primed pluripotency (Brons et al., 2007; Tesar et al., 2007). Interestingly, the late-stage diapause epiblast (EDG9.5 onward) assumes a polarized pattern despite retaining naïve pluripotency networks (Fu et al., 2014; Fan et al., 2020). Fan et al. (2020) recently showed that transient Wnt activity during early stages of mouse diapause (peaking at EDG7.5 and downregulated by EDG9.5) is required to delay polarization and retain naïve pluripotency of the Epi cells. Polarization is prevented via the naïve pluripotency TF Essrb. The epiblast of Esrrb null blastocysts is not maintained during prolonged diapause (Fan et al., 2020). Wnt pathway activity thus promotes naïve pluripotency and prevents polarization in ES cells, as well as in the diapause epiblast, despite its dispensability in normal blastocysts. These observations indicate that establishment of diapause is thus an active process that entails both transcriptional rewiring and morphological alterations. Open questions that remain unanswered are how naïve pluripotency is retained once the epiblast polarizes at EDG9.5 and whether other naïve pluripotency factors are indispensable for epiblast maintenance during diapause.

Wnt pathway activity controls cell polarity by modulating the activities of ROCK and JNK kinases, which control cellular cytoskeleton structure including e.g., actin polymerization. In addition, β-catenin is required for cell adhesion by connecting E-cadherin and α-catenin (Drees et al., 2005). Following the trend for general Wnt activity, β-catenin is also dispensable for normal blastocyst formation (Haegel et al., 1995; Huelsken et al., 2000). Yet, β-catenin null blastocysts collapse upon induction of diapause (before EDG5.5) (Fan et al., 2020). This more severe phenotype compared to Esrrb KO suggests the involvement of cellular adhesion pathways in establishing the diapause state (Fan et al., 2020). Indeed, several genomic profiling studies comparing diapause and active blastocysts documented altered expression of cellular adhesion genes (Hamatani et al., 2004; Fu et al., 2014; van der Weijden et al., 2019). Adhesion pathways and the cytoskeleton are not merely structural components of the cell, but also affect transcriptional networks, as they are additionally involved in signaling pathways and chromatin regulation (Klages-Mundt et al., 2018; Griffith et al., 2021). Thus, altered adhesion properties may have an impact on the diapause blastocyst at multiple levels, e.g., by altering implantation capacity of the TE and polarity of the epiblast, by maintaining the blastocoel and adjusting the blastocyst in its new elongated form, and by altering transcriptional networks to adapt cellular states. Detailed analysis on how adhesion, mechanotransduction, and cytoskeleton pathways impact stem cell states in diapause are missing to date.

### Post-transcriptional and Post-translational Gene Regulation in Diapause

Post-transcriptional mechanisms, such as miRNA-based gene control, are critical regulators of gene activity. Indeed, only about 40% of gene activity is explainable by nascent transcription, although the exact fraction is cell-type dependent (Buccitelli and Selbach, 2020). MicroRNAs (miRNA) are small non-coding RNAs that largely repress gene activity via transcriptional silencing, transcript degradation or translation inhibition (Treiber et al., 2019). MiRNA-mediated gene repression combines sequence specificity with promiscuity (due to short seed sequence and by allowing mismatches), thereby allowing control of multiple target genes at once (O’Brien et al., 2018). Combinatorial miRNA activity, i.e., multiple miRNAs controlling a given gene and one miRNA controlling multiple genes, provides pathway-level control (Bartel and Chen, 2004) and thus is suited to mediate cell state shifts that entail alterations of many pathways. Furthermore, miRNAs usually control metabolic, proliferation, apoptosis, and developmental pathways, all of which are essential components of diapause. As such, miRNAs are potential prominent regulators of diapause and therefore have attracted attention. As miRNA activity fine-tunes gene expression and increases the robustness of genetic programs, miRNA-mediated control of diapause likely acts in combination with other regulatory units to achieve the final outcome (Bartel and Chen, 2004).

The evidence for miRNA function in diapause largely originates from genomic profiling studies. Diapause results in altered miRNA profiles in many mammalian and non-mammalian species (silk worms (Fan et al., 2017), mosquito (Meuti et al., 2018), *C*. *elegans* (Meuti et al., 2018), mouse (Liu et al., 2012). It is therefore possible that distinct miRNAs mediate diapause induction, maintenance, and reactivation. Cell-type specific miRNAs may mediate tissue specificity. For example, in *C. elegans* diapause, the miRNA miR-71 suppresses the growth-promoting PI3K/insulin pathway and miR-235 acts downstream of the insulin pathway to arrest development, pointing at the central role of the insulin pathway in diapause control across species. Knock-outs of these miRNAs are not lethal in diapause, suggesting auxiliary roles in dormancy transition. In the mouse, overexpression of let-7, among the first miRNAs to be discovered in *C. elegans* and a major regulator of developmental timing, induces *ex vivo* diapause by suppressing mTOR and Myc activities and by inhibiting polyamine synthesis (Liu et al., 2020). In mouse diapause, let-7 is of maternal origin and is transferred to the embryo via extracellular vesicles (Figure 2), exemplifying direct maternal control of the embryonic state (Liu et al., 2020). The authors state that let-7 can also induce diapause in human embryos, however, the duration and rate of pausing is marginal (day 7 survival 52% vs. 30%, day 8 survival 5% vs. 0% in let-7 vs. control embryos).

Given the promise of miRNA control over diapause, it is desirable to dissect spatial and temporal regulation by combinatorial action of miRNAs. Mechanistically, miRNA targets are easy to predict but hard to precisely define, since sequence complementarity may not suffice. Studies focusing on individual miRNAs would benefit from identification of miRNA targets. Dissection of downstream pathways can enhance our understanding of *how* miRNAs regulate diapause. MiRNAs are often in feedback control with cellular pathways and active miRNA levels are regulated either transcriptionally, post-transcriptionally (with selective processing), or via sequestration of mature miRNAs, thus acute functional perturbations are necessary to dissect the regulatory complexity of miRNA activity (Treiber et al., 2019).

Many miRNAs have oncogenic activity (Dhawan et al., 2018). Given the importance of understanding dormancy pathways in cancer, investigating miRNA-mediated dormancy in diapause may enlighten oncogenic miRNA activity. miRNAs are assembled into extracellular vesicles and circulating oncogenic miRNAs are used as cancer biomarkers (Hou et al., 2015). It is an intriguing possibility to use circulating miRNAs as potential diapause biomarkers, especially in wildlife species where diapause detection has otherwise not been successful.

Among other post-transcriptional or -translational regulators of pluripotency maintenance in diapause are mRNA stability, alternative splicing, and protein localization. Cnot3, a deadenylator that controls RNA stability, is required to maintain the pluripotent epiblast during mouse diapause by targeting mRNAs of differentiation genes for degradation (Zheng et al., 2016). Cnot3 is not required in normal blastocysts and its deletion is only embryonic lethal at ∼E6.5, thereby illustrating the occurrence of another regulator required for prolonged pluripotency maintenance in ES cells and in diapause (Zheng et al., 2016). Lkb1, an upstream regulator of the starvation-induced AMPK kinase, is controlled by alternative splicing during diapause (Hussein et al., 2020). Other genes such as the developmental TF Pitx1, which also regulates prolactin expression, are also subject to alternative splicing in diapause (Hussein et al., 2020). Finally, the Forkhead family TFs Foxo1/3/4 are associated with dormant states in *C. elegans* (Sun et al., 2017), tammar wallaby, and mink and may control diapause via altered cellular localization in response to altered PI3K/insulin signaling (Fenelon et al., 2017). Although Foxo/Daf-16 has been established as a master regulator of dauer diapause in *C. elegans*, evidence of mammalian Foxo activity in diapause is largely based on stainings and requires functional perturbations (Sun et al., 2017). Foxo function is dispensable for normal development (Kuscu et al., 2019), but is essential to maintain pluripotency in mouse and human ES cells (Zhang et al., 2011), as well as tissue stem cells. This suggests that it may be another factor specifically required to maintain prolonged pluripotency and may be a common inducer of dormancy. Foxo involvement in metabolism, cell cycle, DNA repair control, and protection against oxidative stress (Kops et al., 2002; Fenelon et al., 2017) are highly relevant for diapause, and suggest it may be a master regulator of dormancy.

## Outlook

In this review we highlight the complex biology of diapause that we are only beginning to understand. Current evidence supports a model in which diapause is initially triggered due to the embryo’s inability to implant into the non-receptive uterus. Entry into diapause is followed by morphological, metabolic, and genomic restructuring of the embryo as well as maternal tissues. Soluble and vesicle-delivered factors in the uterine microenvironment mediate maternal-embryo crosstalk and control diapause. Absence of key metabolites, e.g., polyamines, induce diapause and presence of others, e.g., lipids, sustain embryos in diapause. Key pluripotency-maintenance factors, e.g., LIF, are required to maintain the epiblast throughout diapause. Diapause-like states can be induced *ex vivo*, e.g., via mTOR and Myc inhibitors or miRNA supplementations. Taken together, diapause is induced through multiple routes and entails complex restructuring of embryonic and extraembryonic networks.

Current challenges in further investigation of diapause include technical limitations in embryo accessibility, recovery, and manipulation. These challenges illustrate the need for *ex vivo* and *in vitro* diapause model systems. Spatial and temporal regulation can only be understood by designing dynamic experiments and by addressing inter-embryo heterogeneity and cell-type specificity. Implementing single-cell transcriptome and accessibility profiling methods will greatly enhance our understanding of spatiotemporal regulation. Recovering normal or diapause embryos in many wild animals is not feasible and identification of diapause biomarkers would facilitate reproductive technology in these species. ES pluripotency pathways are rooted in prolonged pluripotency maintenance in diapause, thus studying diapause could enable establishment of ES or iPS cells from so-far refractive species.

## Author Contributions

VvdW and AB-K conceptualized and co-wrote the manuscript. Both authors contributed to the article and approved the submitted version.

## Conflict of Interest

The authors declare that the research was conducted in the absence of any commercial or financial relationships that could be construed as a potential conflict of interest.

## Publisher’s Note

All claims expressed in this article are solely those of the authors and do not necessarily represent those of their affiliated organizations, or those of the publisher, the editors and the reviewers. Any product that may be evaluated in this article, or claim that may be made by its manufacturer, is not guaranteed or endorsed by the publisher.
